# Adsorption of magnetic manganese ferrites to simulated monomeric mercury in flue gases

**DOI:** 10.1371/journal.pone.0304333

**Published:** 2024-06-14

**Authors:** Lei Sun, Xiajun Zhang, Zhou Wang, Min Liu

**Affiliations:** 1 Danyang Maternal and Child Health Hospital, Zhenjiang, P.R. China; 2 The People’s Hospital of Danyang, Affiliated Danyang Hospital of Nantong University, Zhenjiang, P.R. China; 3 Vanadium and Titanium Resource Comprehensive Utilization Key Laboratory of Sichuan Province, School of Vanadium and Titanium, Panzhihua University, Panzhihua, P.R. China; Hamadan University of Medical Sciences, ISLAMIC REPUBLIC OF IRAN

## Abstract

Magnetic MnFe_2_O_4_ nanoparticles were successfully prepared by the rapid combustion method at 500 °C for 2 h with 30 mL absolute ethanol, and were characterized by SEM, TEM, XRD, VSM, and XPS techniques, their average particle size and the saturation magnetization were about 25.3 nm and 79.53 A·m^2^/kg, respectively. The magnetic MnFe_2_O_4_ nanoparticles were employed in a fixed bed experimental system to investigate the adsorption capacity of Hg^0^ from air. The MnFe_2_O_4_ nanoparticles exhibited the large adsorption performance on Hg^0^ with the adsorption capacity of 16.27 μg/g at the adsorption temperature of 50 °C with the space velocity of 4.8×10^4^ h^-1^. The VSM and EDS results illustrated that the prepared MnFe_2_O_4_ nanoparticles were stable before and after adsorption and successfully adsorbed Hg^0^. The TG curves demonstrated that the mercury compound formed after adsorption was HgO, and both physical and chemical adsorption processes were observed. Magnetic MnFe_2_O_4_ nanoparticles revealed excellent adsorbance of Hg^0^ in air, which suggested that MnFe_2_O_4_ nanoparticles be promising for the removal of Hg^0^.

## 1. Introduction

Mercury is harmful to ecosystems because of its long-range transport, persistence, and bioaccumulation [1–3]. Mercury can undergo various stages of transformation to produce methylmercury (MeHg), a highly toxic form of mercury, the ingestion of which can have adverse effects on human health [4]. The increasing concentration of mercury in the environment has attracted the attention of governments and environmental organizations, and has become a worldwide environmental problem. The Minamata Convention on mercury, which entered into force in 2017, emphasizes cost-effective abatement and efficient decontamination of mercury pollution, as well as the sound management and utilization of mercury-rich waste [5]. Coal-fired power plants are reported to be the most significant source of mercury emissions, so in response to this problem, the Emission Standards for Air Pollutants from Power Plants (GB 132232011), published in July 2011, for the first-time limit mercury emission concentrations to no more than 0.03 mg/m^3^ of mercury and mercury compounds from coal-fired power plants [6].

Mercury in coal combustion flue gas exists in three main forms: elemental mercury (Hg^0^), oxidized mercury (Hg^2+^), and particulate bound mercury (Hg^p^) [7–9]. Hg^2+^ is water soluble and volatile, can be readily adsorbed and removed via a wet scrubber process. Hg^P^ is susceptible to adsorption and charging, it has a short residence time in the atmosphere, and it is easily removed by electric precipitators. Since Hg^0^ is highly volatile and almost insoluble in water, where it remains in the atmosphere for 0.5–2 years. More importantly, Hg^0^ is one of the most difficult form to control, and it is challenging to remove mercury from coal-fired flue gases [10].

Catalysis and adsorption methods are the main methods for the removal of elemental mercury [11–14]. In recent years, various catalysts and adsorbents have been reported for mercury removal, such as activated carbon, precious metals, and metal oxides [8, 15, 16]. Among them, magnetic ferrite nanomaterials are a new type of material that offers the advantages of easy separation, recyclability, and environmental friendliness, so they have been widely applied in organic and biological separations. Especially, they are excellent adsorbents due to their large specific surface area, abundant active sites, and interfacial effect [17–19]. Simultaneously, manganese oxides can impact the mercury cycle by adsorbing it, influencing the ambient redox potential and regulating microorganism activity. Therefore, MnFe_2_O_4_ is recognized as an excellent adsorbent for removing singlet Hg from coal-fired flue gases. To enhance the removal performance of MnFe_2_O_4_ nanoparticles, researchers have proposed several constructive ideas, such as applying organic or inorganic layers to coat the surface of nanomaterials [20–22], modifying the structural composition and morphology parameters [23], and doping transition metals [24].

Various approaches are available for the preparation of magnetic nanomaterials, such as co-precipitation [25], hydrothermal methods [26], rapid combustion methods [27], and so on. The birdnesting and the nonuniformity of composition are the largest problem owing to the accession of precipitant. While, magnetic nanoparticles prepared by hydrothermal method have long cycle time and small yield. The rapid combustion method has a short preparation cycle, low cost, safe, reliable, and environmentally friendly process, it is easy to produce industrially, and it can achieve the purpose of controlling the sizes and properties of magnetic nanoparticles by changing the amount of solvent and calcination temperature [28–30]. And the application of adsorbents prepared by this method for the removal of elemental mercury from coal combustion flue gas has not been reported.

In this paper, magnetic MnFe_2_O_4_ nanomaterials were prepared from metal nitrates by a rapid combustion method based on the ability of manganese compounds to oxidize mercury, and the factors (volume of anhydrous ethanol and calcination temperature) affecting their adsorption properties were optimized. The mercury removal performance of the magnetic MnFe_2_O_4_ nanomaterials was evaluated in a fixed bed system. Combined with vibrating sample magnetometry (VSM), energy dispersive spectroscopy (EDS) mapping, and thermogravimetric (TG) curves, the mercury removal process was analyzed.

## 2. Experiment details

### 2.1 Preparation and characterization of magnetic MnFe_2_O_4_ nanoparticles

Magnetic MnFe_2_O_4_ nanoparticles were prepared via the rapid combustion process, typically, Fe(NO_3_)_3_·9H_2_O and Mn(NO_3_)_2_·6H_2_O were dissolved into 30 mL absolute ethanol according to 1:2 molar ratio of them to form a homogeneous solution, ignited and burned until extinguished, and calcinated at 400–800 °C for 2 h with the heating rate of 3 °C/min to obtain magnetic MnFe_2_O_4_ nanoparticles. In the rapid combustion process, anhydrous ethanol acted as both a dispersant and a fuel, effectively dispersing the solute while serving as a catalyst for combustion. The product obtained at the end of the combustion of the solution incompletely formed MnFe_2_O_4_ crystals in a sol state. Subsequently, the semi-finished product was transferred to the temperature-controlled furnace programmed for high-temperature calcination. The purpose of this process was to provide sufficient heat for inducing the formation of MnFe_2_O_4_ nanoparticles and removed impurities such as activated carbon and nitrate.

The microscopic morphology and elemental distribution of MnFe_2_O_4_ nanomaterials were characterized by scanning electron microscopy (SEM), transmission electron microscopy (TEM), and energy dispersive spectroscopy (EDS). The physical phase and crystallinity were analyzed through X-ray diffractometer (XRD). Vibrating sample magnetometer (VSM) was employed to measure the hysteresis loops of MnFe_2_O_4_ nanoparticles before and after adsorption. The formation of the products in the adsorption process was determined by a thermogravimetric curve (TG).

### 2.2 Elemental mercury adsorption experiments

**Fig 1:**
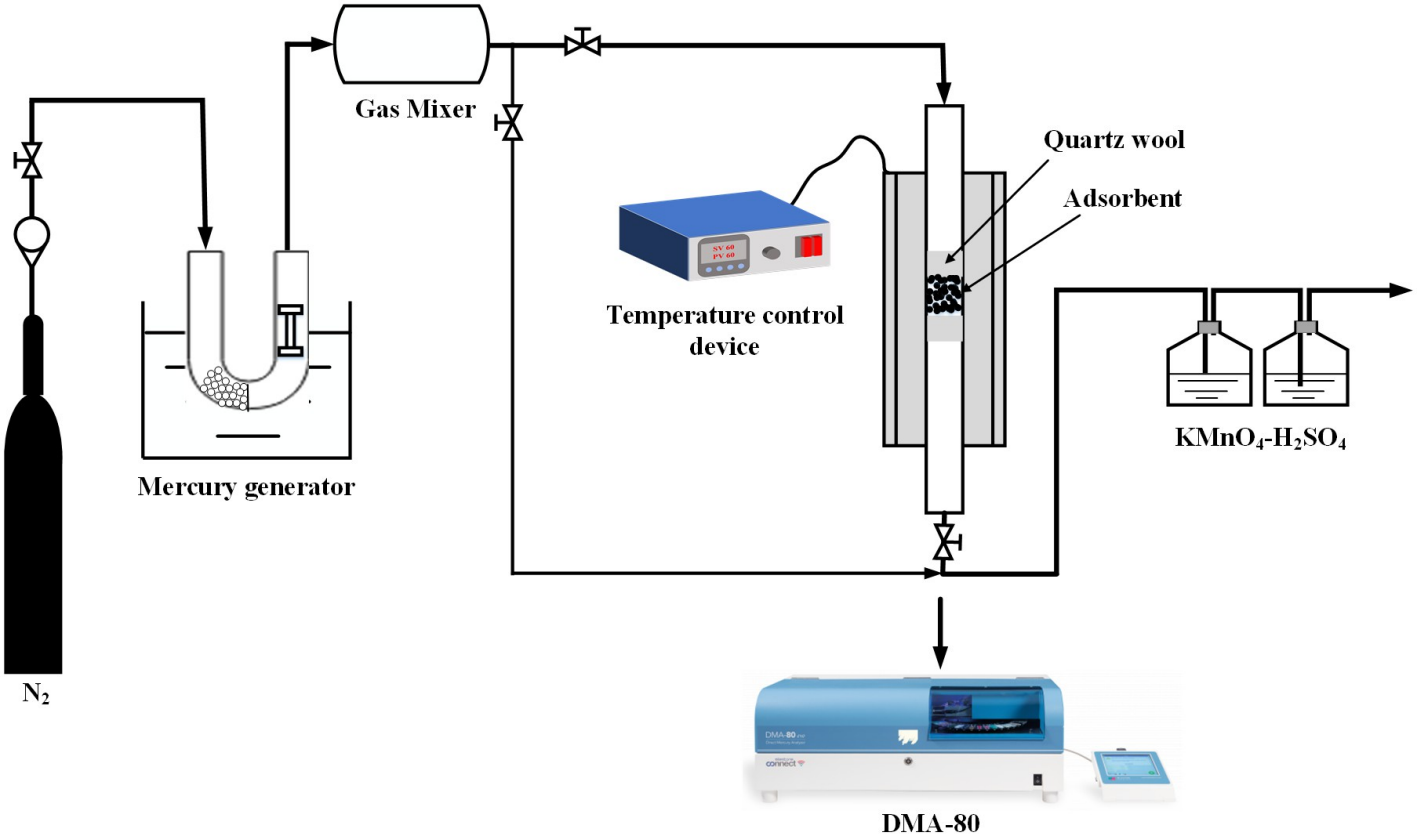
Simple diagram of a fixed bed experimental setup.

The actual coal combustion flue gas contains a variety of gases such as N_2_, CO_2_, O_2_, and SO_2_. The influence of the various gases on the removal of mercury was complex, and considering that most of the flue gas was N_2_, only N_2_ was chosen for the experiments (Fig 1). The gas flow rate was controlled by a rotameter and adjusted according to the experimental requirements. Various concentrations of mercury vapors were produced by means of a heated mercury permeation tube. The adsorbent was packed in a U-shaped tube with an inner diameter of 5 mm. To prevent the adsorbent from falling or being blown up by the gas, cotton was inserted at both ends for support and fixation. All experimental piping and connections were made of poly tetra fluoroethylene (PTFE). The tail gas after the reaction is purified with 10% H_2_SO_4_-4% KMnO_4_ absorption solution and discharged. Once adsorbed, the solid nanoparticles were detected by means of DMA-80 mercury analyzer. The experimental data were obtained after three experiments.

## 3. Results and discussion

### 3.1 Characterization of magnetic MnFe_2_O_4_ nanoparticles

**Fig 2:**
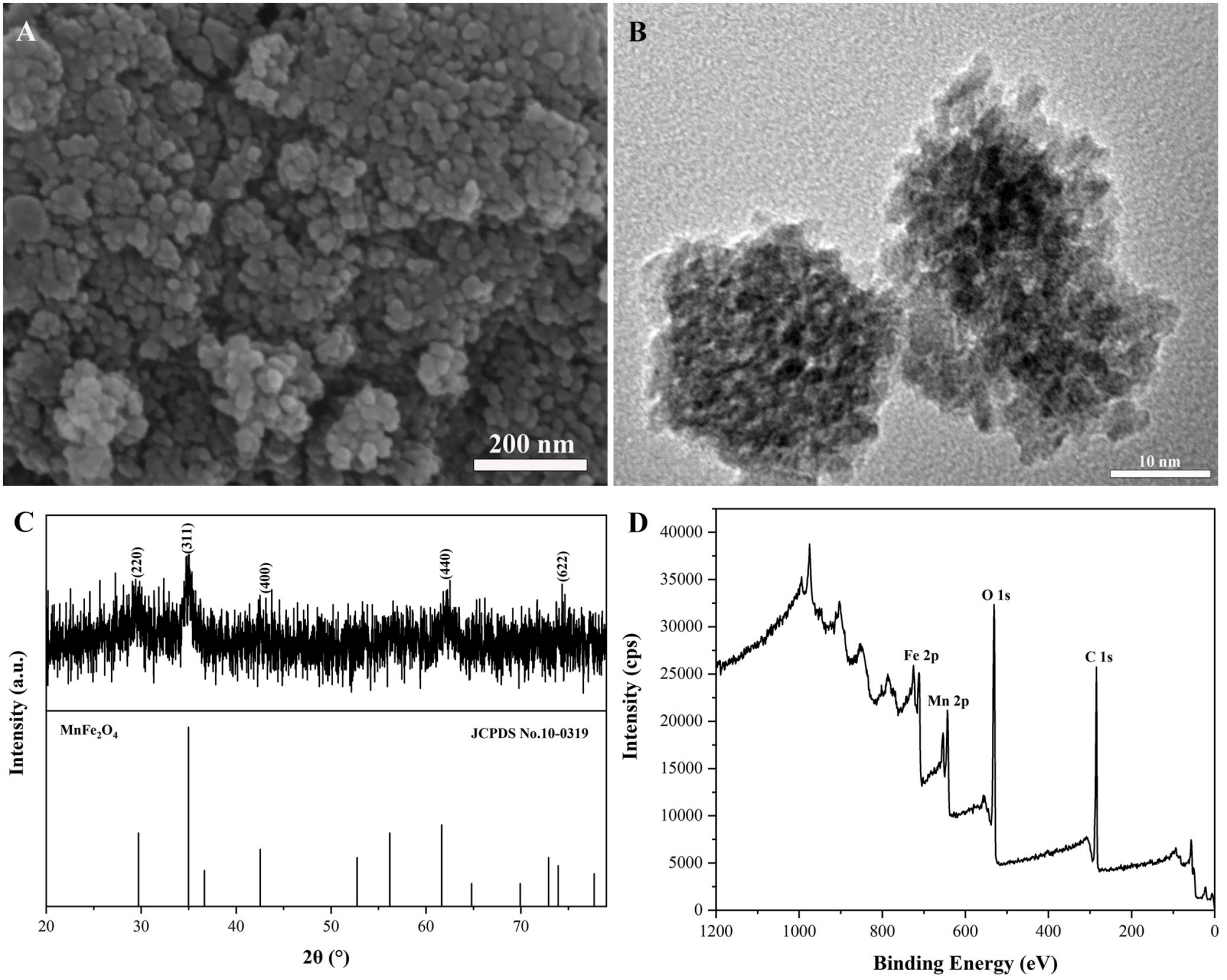
(A) SEM morphology, (B) TEM image, (C) XRD pattern, and (D) XPS spectra of magnetic MnFe_2_O_4_ nanoparticles obtained by calcination at 500 °C for 30 mL of absolute ethanol for 2 h.

Fig 2 showed the SEM morphology, TEM image, XRD pattern, and XPS spectra of the MnFe_2_O_4_ nanomaterials calcinated at 500 °C for 2 h with 30 mL absolute ethanol. As shown in Fig 2A (S1 Fig), the morphology of the prepared MnFe_2_O_4_ nanomaterials exhibited a granular shape with an average particle size of about 25.3 nm. The TEM image was shown in Fig 2B (S2 Fig), the measured morphology was granular and the average particle size was also around 25.3 nm, which was in general agreement with the observation from SEM morphology. The excellent nano-size made the adsorption capacity extraordinarily large, making it an excellent choice for absorbing Hg^0^. XRD pattern of MnFe_2_O_4_ nanoparticles was shown in Fig 2C (S3 Fig), the characteristic peaks were 29.62°, 34.82°, 43.13°, 62.29°, and 74.34°, corresponding to the (220), (311), (400), (440), and (622) crystalline planes of the MnFe_2_O_4_ standard card (JCPDS No. 10–0319), which proved that the MnFe_2_O_4_ nanoparticles were prepared successfully [31]. The prepared nanomaterials possessed four components, Mn, Fe, O, and C, as could be seen from the XPS maps (Fig 2D, S4 Fig), which again demonstrated the successful preparation of MnFe_2_O_4_ nanoparticles.

### 3.2 Effects of preparation factors for MnFe_2_O_4_ nanoparticles on Hg^0^ adsorption

To understand the effect of MnFe_2_O_4_ nanoparticles prepared with different alcohol volumes and calcination temperatures on the performance of the mercury removal, it was investigated by a fixed bed experimental system [32].

**Fig 3:**
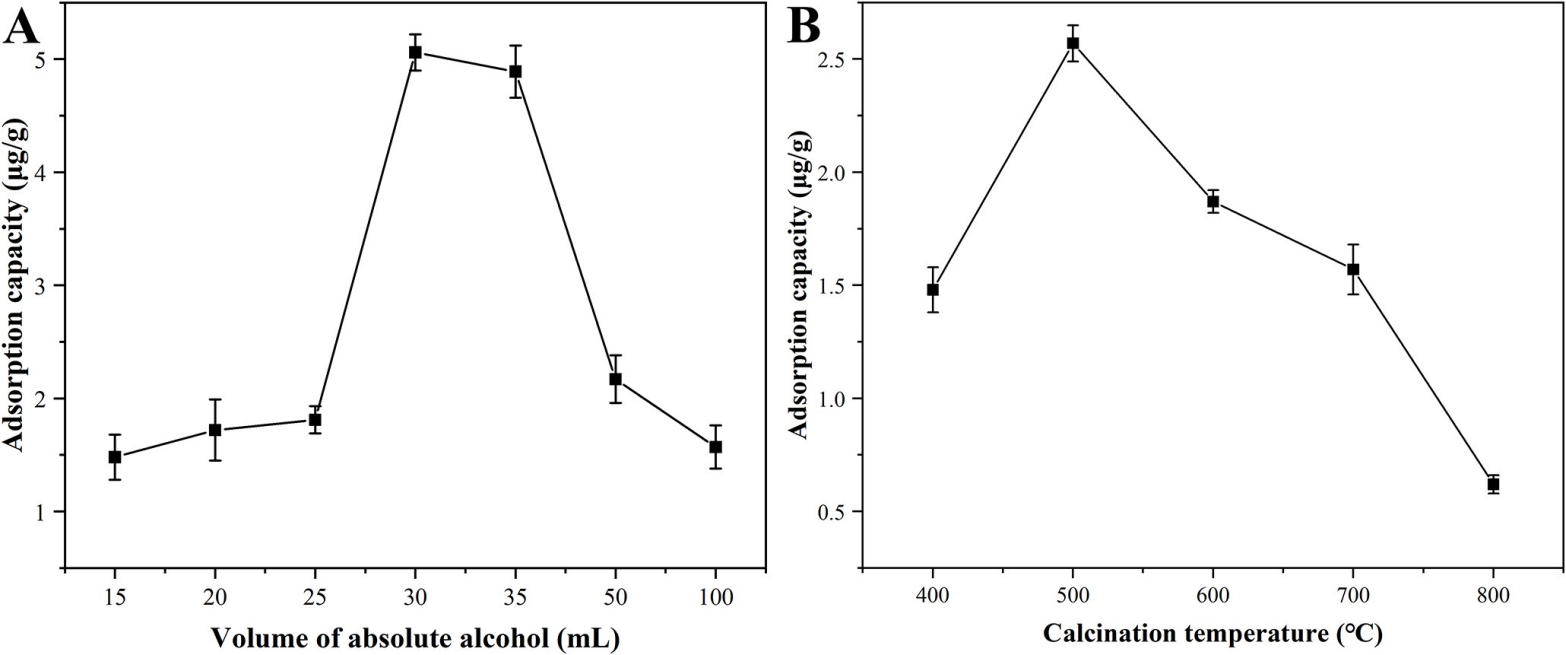
Adsorption curves of Hg^0^ by MnFe_2_O_4_ nanoparticles prepared at different calcination temperatures (B) with various alcohol volumes (A) under permeation temperature of 40 °C, space velocity of 4.8×10^4^ h^-1^, and adsorption temperature of 30 °C.

Anhydrous ethanol was essential in the rapid combustion process, which not only played a role in distributing to the solute and ignition, but also effectively controlled the duration of combustion. As could be seen from Fig 3A (S1 Table), when experimental conditions were controlled at permeation temperature of 40 °C, space velocity of 4.8×10^4^ h^-1^, and adsorption temperature of 30 °C, the adsorption capacity of Hg^0^ increased slowly with the volume of alcohol for the preparation of MnFe_2_O_4_ nanoparticles increasing of from 15 mL to 25 mL, and when the absolute alcohol volume rose to 30 mL, a significant increase of the adsorption capacity could be seen. The reason for this phenomenon might be that the large volume of anhydrous ethanol caused the solute to be dispersed excessively in the solvent, resulting in the formation of smaller grain sizes. However, as the volume of anhydrous ethanol increased further, the adsorption capacity tended to decrease. This might be attributed that the large amount of anhydrous ethanol dispersed the solute, while also prolonging the combustion time. As a result, there was a greater degree of sintering, leading to larger grain size and decreases in specific surface area and adsorption capacity [33]. Therefore, 30 mL was chosen as the optimum volume of absolute alcohol.

Fig 3B (S2 Table) showed the adsorption capacities of MnFe_2_O_4_ nanoparticles prepared at different calcination temperatures for Hg^0^ under permeation temperature of 40 °C, space velocity of 4.8×10^4^ h^-1^, and adsorption temperature of 30 °C. As could be seen from the figures, the adsorption capacity increased with the increase of calcination temperature, reached the maximum adsorption capacity of 2.57 μg/g for MnFe_2_O_4_ nanoparticles calcinated at 500 °C, and then reduces. From previous experience, it could be seen that the crystallinity of the produced nanoparticle was poor at 400 °C of calcination temperature. And when the calcination temperature was increased to 500 °C, the crystallinity improved greatly and the grain size was relatively small. The growth rate of the grains increased with the increase of the calcination temperature, and the crystallinity and grain size also increased, which in turn led to a decrease of specific surface area and a reduction of adsorption capacity [30]. Therefore, 500 °C was chosen as the optimum calcination temperature. In summary, the MnFe_2_O_4_ nanoparticles were prepared with alcohol volume of 30 mL and a calcination temperature of 500 °C for subsequent adsorption experiments.

### 3.3 Influence of adsorption temperature on the performance of Hg^0^ removal

**Fig 4:**
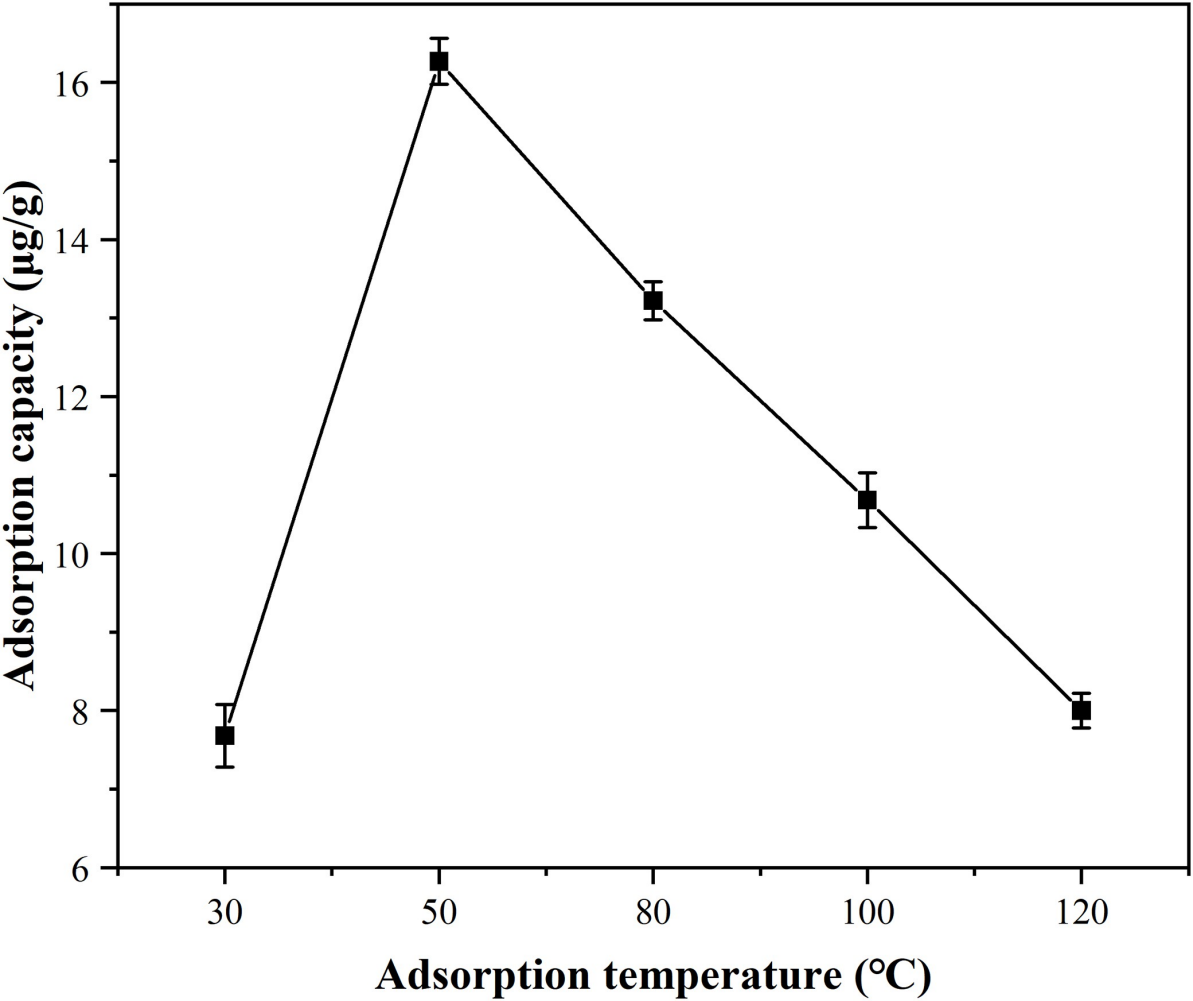
Adsorption capacity of MnFe_2_O_4_ nanoparticles for Hg^0^ at different adsorption temperatures under permeation temperature of 40 °C and space velocity of 4.8×10^4^ h^-1^.

As could be seen from Fig 4 (S3 Table), when the permeation temperature and space velocity were controlled at 40 °C and 4.8×10^4^ h^-1^, respectively, the adsorption capacity of MnFe_2_O_4_ nanoparticles for Hg^0^ increased from 7.68 μg/g to 16.27 μg/g at the adsorption temperature of 30–50 °C. With the further rise of the adsorption temperature, the adsorption capacity of Hg^0^ began to decline, and the adsorption capacity was only 8.0 μg/g at the adsorption temperature of 120 °C. This could be due to the fact that the adsorption of Hg^0^ by MnFe_2_O_4_ nanoparticles was both physical and chemical adsorption mechanism, and the high temperature would cause the desorption of adsorbed Hg^0^, and decrease the adsorption capacity, so the high temperature was not conducive to the physical adsorption. Therefore, the temperature of 50 °C was the optimum temperature for adsorption.

### 3.4 Effect of space velocity on the performance of mercury adsorption

**Fig 5:**
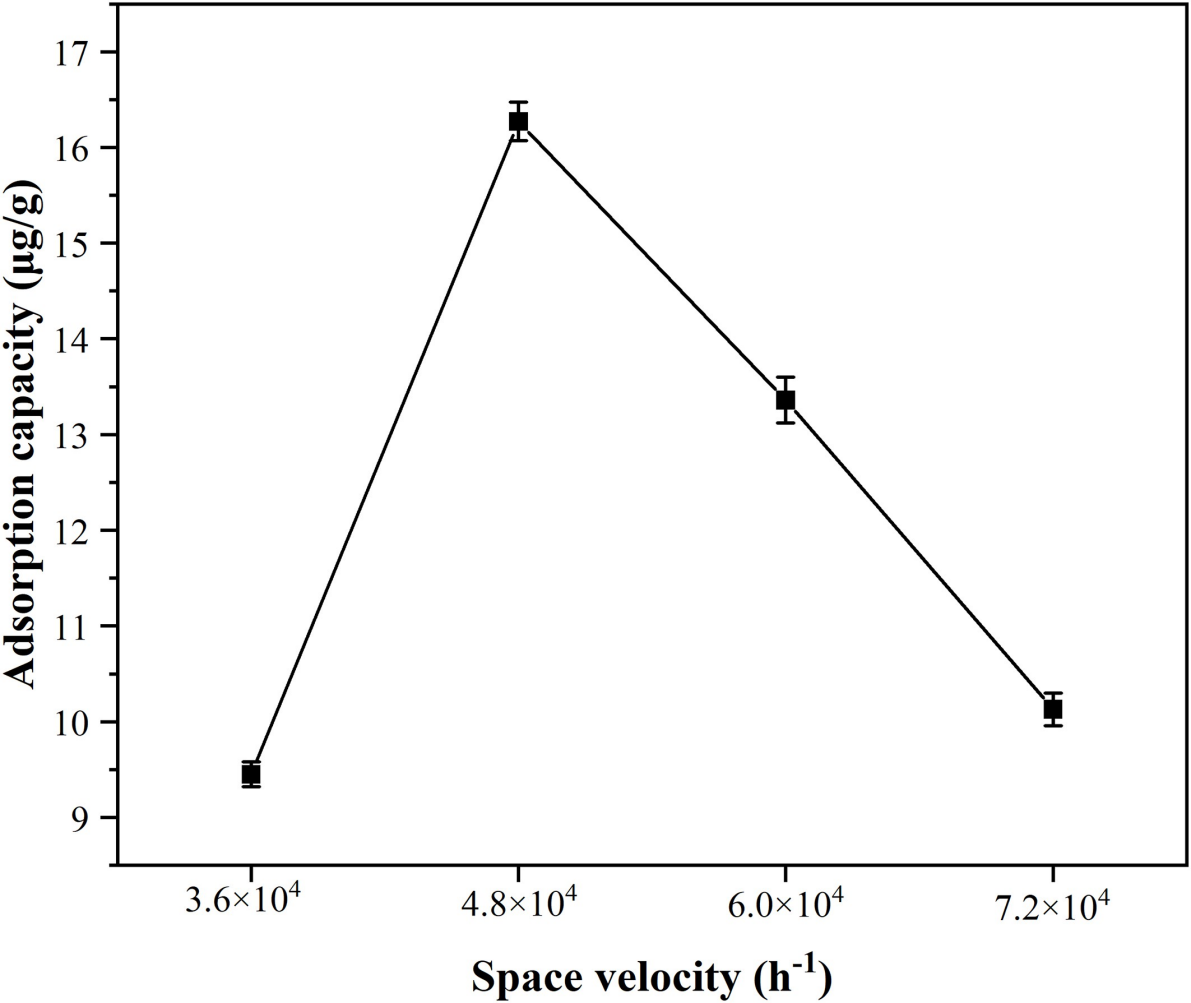
Effect of the space velocity on the performance of MnFe_2_O_4_ nanoparticles for Hg^0^ removal under permeation temperature of 40 °C and the adsorption temperature of 50 °C.

In this experiment, the space velocity was varied by adjusting the flow rate of the simulated flue gas and other experimental conditions were kept constants. When the permeation temperature and the adsorption temperature were controlled at 40 °C and 50 °C, respectively, four gas flow rates (30 mL/min, 40 mL/min, 50 mL/min, and 60 mL/min) were set, and four space velocities were obtained by calculating 3.6×10^4^ h^-1^, 4.8×10^4^ h^-1^, 6.0×10^4^ h^-1^, and 7.0×10^4^ h^-1^ via equation. The adsorption capacities of MnFe_2_O_4_ nanoparticles for Hg^0^ at different space velocities were revealed in Fig 5 (S4 Table). It could be seen from the figure that, when the space velocity was increased from 3.6×10^4^ h^-1^ to 4.8×10^4^ h^-1^, a large increase of the adsorption capacity for Hg^0^ onto MnFe_2_O_4_ nanoparticles occurred. A reduction of adsorption capacity began to occur when the space velocity was greater than 4.8×10^4^ h^-1^, which indicated that the space velocity affected the performance of MnFe_2_O_4_ nanoparticles for the removal of mercury. The simulated flue gas would have a reduced contact time with the MnFe_2_O_4_ nanoparticles due to the rise in space velocity, and the chances of the MnFe_2_O_4_ nanoparticles capturing Hg^0^ would be reduced, thus leading to a decline of adsorption capacity. Therefore, an airspeed lower than 4.8×10^4^ h^-1^ was chosen to be more favorable for Hg^0^ capture by MnFe_2_O_4_ nanoparticles.

### 3.5 Variation in adsorption capacity at different mercury permeation temperatures

**Fig 6:**
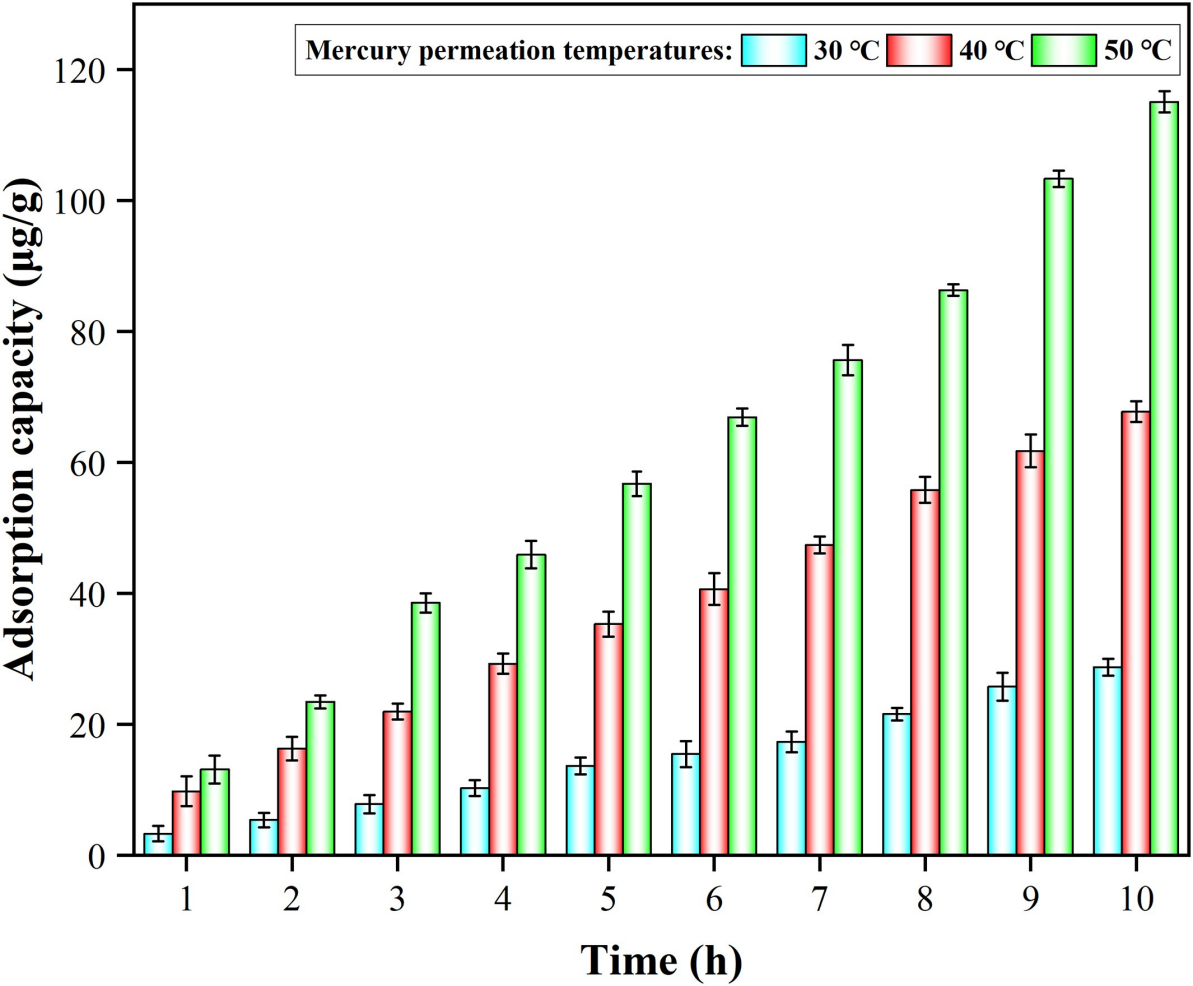
Effect of permeation temperature on Hg^0^ removal by MnFe_2_O_4_ nanoparticles under space velocity of 4.8×10^4^ h^-1^ and adsorption temperature of 50 °C.

Fig 6 (S5 Table) presented a trend of the adsorption capacity of MnFe_2_O_4_ nanoparticles for Hg^0^ at different mercury permeation temperatures under space velocity of 4.8×10^4^ h^-1^ and adsorption temperature of 50 °C. The MnFe_2_O_4_ nanoparticles exhibited a relatively larger adsorption capacity in all three permeation temperature ranges. As the permeation temperature rose, a large increase of adsorption capacity was observed. This was probably attributed to the fact that the increase of the concentration gave the adsorbent an increased opportunity to contact with Hg^0^, which led to an increase of adsorption capacity. And this trend was also important in the practical application of coal-fired power plants, where the amount of adsorbent could be varied according to the actual needs of the plant.

Compared with the previously reported articles related to mercury adsorption in gases (Table 1), the preparation method of MnFe_2_O_4_ nanoparticles proposed in this paper was low cost and easy to operate. In addition, the MnFe_2_O_4_ nanoparticles had better adsorption performance and short adsorption time, the biggest advantage was that MnFe_2_O_4_ could utilize its own magnetism to realize magnetic separation and recycling after adsorption, which effectively avoided secondary pollution.

**Table 1:** Comparison of mercury adsorption from gases by as-prepared MnFe_2_O_4_ nanoparticles with other reported materials.

Adsorbing material	Initial Hg^0^ concentration	Adsorption time (min)	Adsorption performance	References
Powdered activated carbon	500 μg/m^3^	/	0.278 mg/g	[34]
Sulfurizing activate carbon	/	120	1227.5μg/g	[35]
Se/SiO_2_ adsorbent	130 μg/m^3^	14,400	101.04 mg/g	[36]
Fly ash	12.58 μg/m^3^	180	0.005 mg/g	[37]
MnFe_2_O_4_	1.2 mg/m^3^	60	16.27 μg/g	This work

### 3.6 Characterization of MnFe_2_O_4_ nanoparticles before and after adsorption

**Fig 7:**
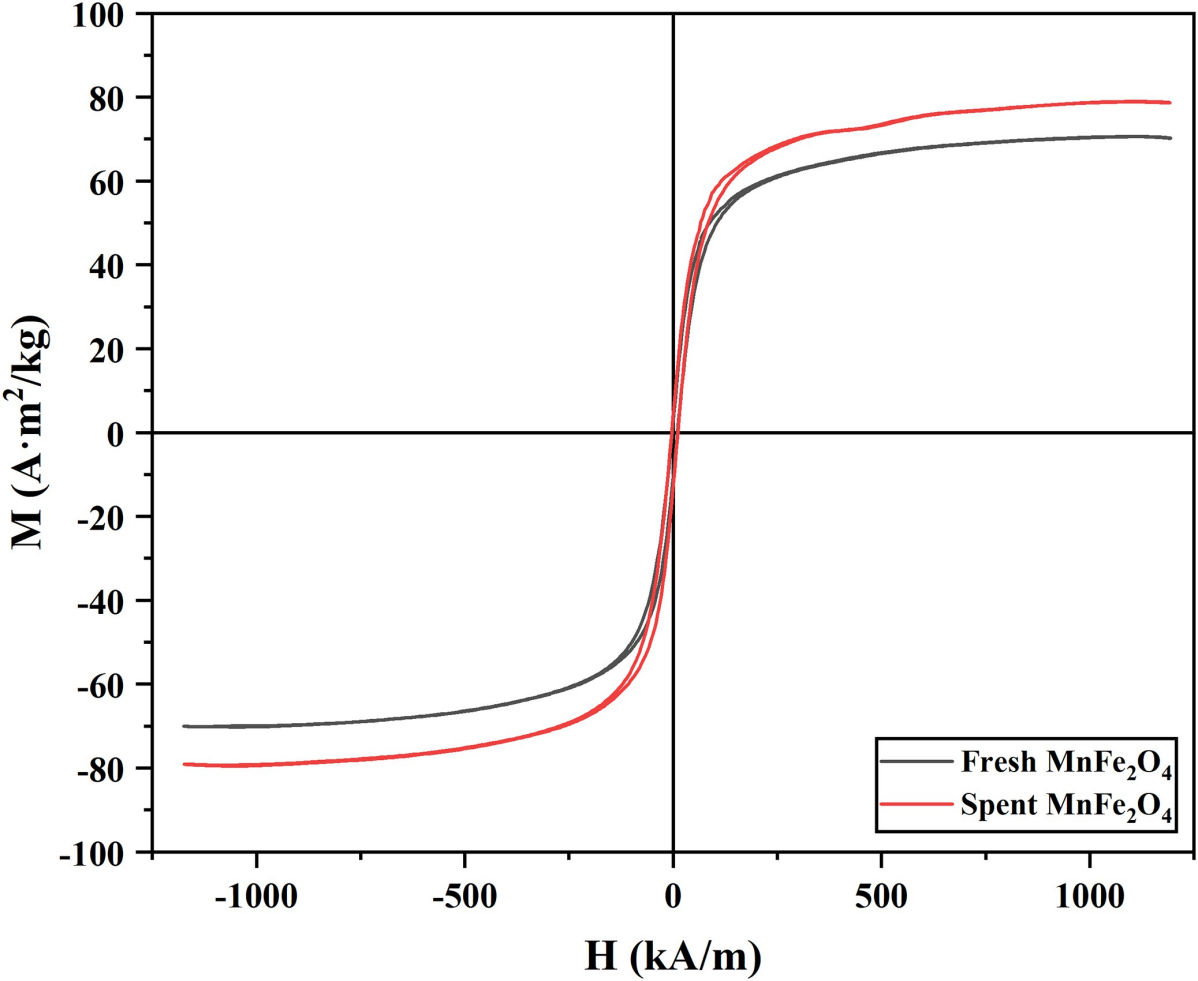
Hysteresis loops of MnFe_2_O_4_ nanoparticles before and after adsorption.

The magnetic properties of the MnFe_2_O_4_ nanoparticles before and after adsorption of Hg^0^ were displayed in Fig 7. Obviously, the saturation magnetization of fresh MnFe_2_O_4_ nanoparticles was 79.53 A·m^2^/kg, after adsorption of Hg^0^, the saturation magnetization intensity of the MnFe_2_O_4_ nanoparticles decreased to 70.21 A·m^2^/kg. Although the magnetic properties of the MnFe_2_O_4_ nanoparticles were slightly reduced, the overall saturation magnetization intensity of the MnFe_2_O_4_ nanoparticles was still very superior, and gas-solid separation could be achieved by magnetic separation, which avoided secondary pollution.

**Fig 8:**
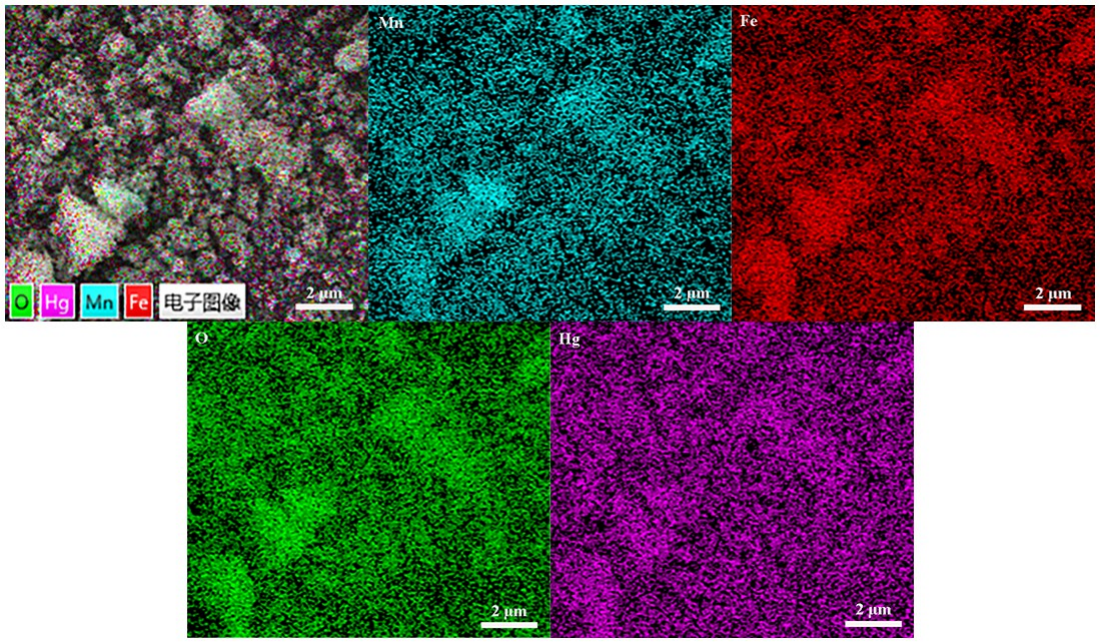
EDS plot of MnFe_2_O_4_ nanoparticles after adsorption.

Fig 8 showed the SEM morphology of the MnFe_2_O_4_ nanoparticles after adsorption of Hg^0^. It could be seen that there was a slight agglomeration of the adsorbed MnFe_2_O_4_ nanoparticles with no significant change in the morphology compared with Fig 1A. This demonstrated that the adsorption of Hg^0^ by MnFe_2_O_4_ nanoparticles did not damage the themselves structure of the nanomaterials, which also indicated the possibility of recycling. The EDS plot revealed that the adsorbed materials contained the four elements Mn, Fe, O, and Hg, which demonstrated that Hg^0^ was successfully adsorbed onto the adsorbent of MnFe_2_O_4_ nanoparticles [38].

### 3.7 TG analyses of MnFe_2_O_4_ nanoparticles before and after adsorption

**Fig 9:**
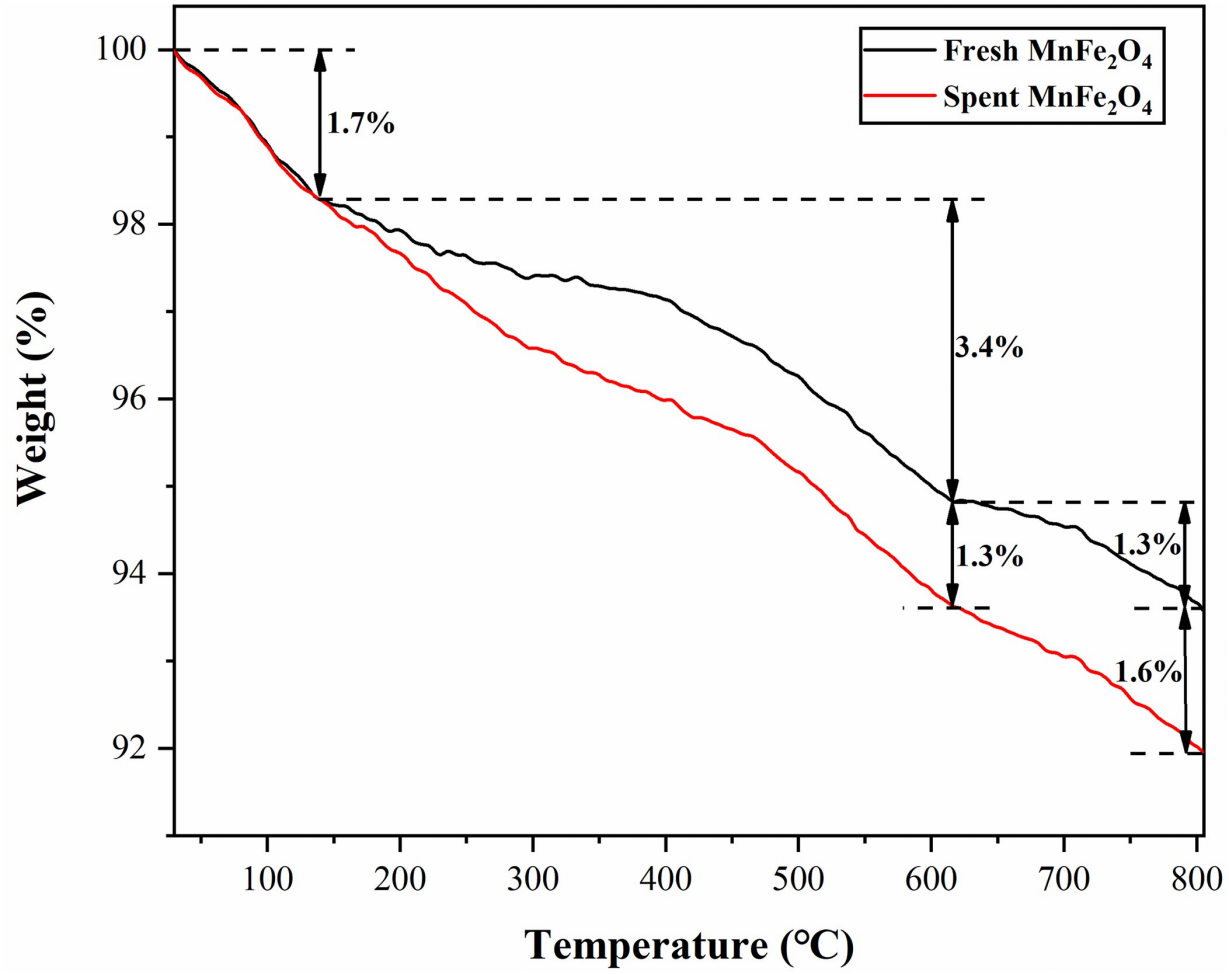
TG curves of the fresh and the spent MnFe_2_O_4_ nanoparticles.

To further investigate the mechanism of Hg^0^ removal before and after adsorption, TG analyses were performed on fresh and spent MnFe_2_O_4_ nanoparticles. As could be seen from Fig 9, the MnFe_2_O_4_ nanoparticles have about 1.7% mass loss below 140 °C, which was attributed to the loss of free water. At 140 °C-614 °C, fresh nanoparticles showed a mass loss of 3.7%, which might be caused by the evaporation of bound water in the nanomaterials and the combustion breakdown of part of the carbon skeleton. The mass loss of the spent nanoparticles was more than 1.3% compared with the fresh nanoparticles, which could be due to the desorption of the adsorbed mercury species. When the temperature exceeded 140 °C, the adsorbed Hg^0^ on the MnFe_2_O_4_ nanoparticles was gradually thermally desorbed out, which indicated the presence of physical adsorption in the adsorption process. A significant mass loss was also observed in the range of 400 °C-600 °C, which was due to the decomposition of HgO, demonstrating the chemisorption of Hg^0^ by the MnFe_2_O_4_ nanoparticles [6, 13, 39].

## 4. Conclusion

In this project, magnetic MnFe_2_O_4_ nanoparticles were prepared by the rapid combustion method, and the effects of absolute alcohol volume and calcination temperature on magnetic properties and average grain size were investigated during the preparation process. The adsorption effects of different experimental conditions on Hg^0^ were examined by a fixed-bed experimental system. The experimental results revealed that the average particle size and the saturation magnetization of MnFe_2_O_4_ nanoparticles prepared at the calcination temperature of 500 °C with 30 mL of absolute ethanol were 25.3 nm and 79.53 A·m^2^/kg, and they exhibited the excellent adsorption capacity of 16.27 μg/g for Hg^0^ at adsorption temperature of 50 °C and a space velocity of 4.8×10^4^ h^-1^. The comparison of the saturation magnetization intensity before and after adsorption displayed the magnetic stability of the MnFe_2_O_4_ nanoparticles after adsorption, which facilitated the separation and reuse in subsequent experiments. EDS and TG plots demonstrated the successful adsorption of Hg^0^ onto the MnFe_2_O_4_ nanoparticles with the formation of the mercury compound as HgO, and the presence of both physical and chemical adsorption.

## Supporting information

S1 Fig SEM morphology of magnetic MnFe_2_O_4_ nanoparticles obtained by calcination at 500 °C for 30 mL of absolute ethanol for 2 h. (TIF)

S2 Fig TEM image of magnetic MnFe_2_O_4_ nanoparticles obtained by calcination at 500 °C for 30 mL of absolute ethanol for 2 h. (BMP)

S3 Fig XRD pattern of magnetic MnFe_2_O_4_ nanoparticles obtained by calcination at 500 °C for 30 mL of absolute ethanol for 2 h. (TIF)

S4 Fig XPS spectra of magnetic MnFe_2_O_4_ nanoparticles obtained by calcination at 500 °C for 30 mL of absolute ethanol for 2 h. (TIF)

S1 Table Adsorption data of Hg^0^ by MnFe_2_O_4_ nanoparticles prepared with various alcohol volumes under permeation temperature of 40 °C, space velocity of 4.8×10^4^ h^-1^, and adsorption temperature of 30 °C. (DOCX)

S2 Table Adsorption data of Hg^0^ by MnFe_2_O_4_ nanoparticles prepared at different calcination temperatures under permeation temperature of 40 °C, space velocity of 4.8×10^4^ h^-1^, and adsorption temperature of 30 °C. (DOCX)

S3 Table Adsorption capacity of MnFe_2_O_4_ nanoparticles for Hg^0^ at different adsorption temperatures under permeation temperature of 40 °C and space velocity of 4.8×10^4^ h^-1^. (DOCX)

S4 Table Effect of the space velocity on the performance of MnFe_2_O_4_ nanoparticles for Hg^0^ removal under permeation temperature of 40 °C and the adsorption temperature of 50 °C. (DOCX)

S5 Table Effect of permeation temperature on Hg^0^ removal by MnFe_2_O_4_ nanoparticles under space velocity of 4.8×10^4^ h^-1^ and adsorption temperature of 50 °C. (DOCX)
