# Supplementary figures and images for: Adsorption of magnetic manganese ferrites to simulated monomeric mercury in flue gases
Source: PLoS One. 2024 Jun 14;19(6):e0304333. doi: 10.1371/journal.pone.0304333 (PMC11178181; doi:10.1371/journal.pone.0304333)

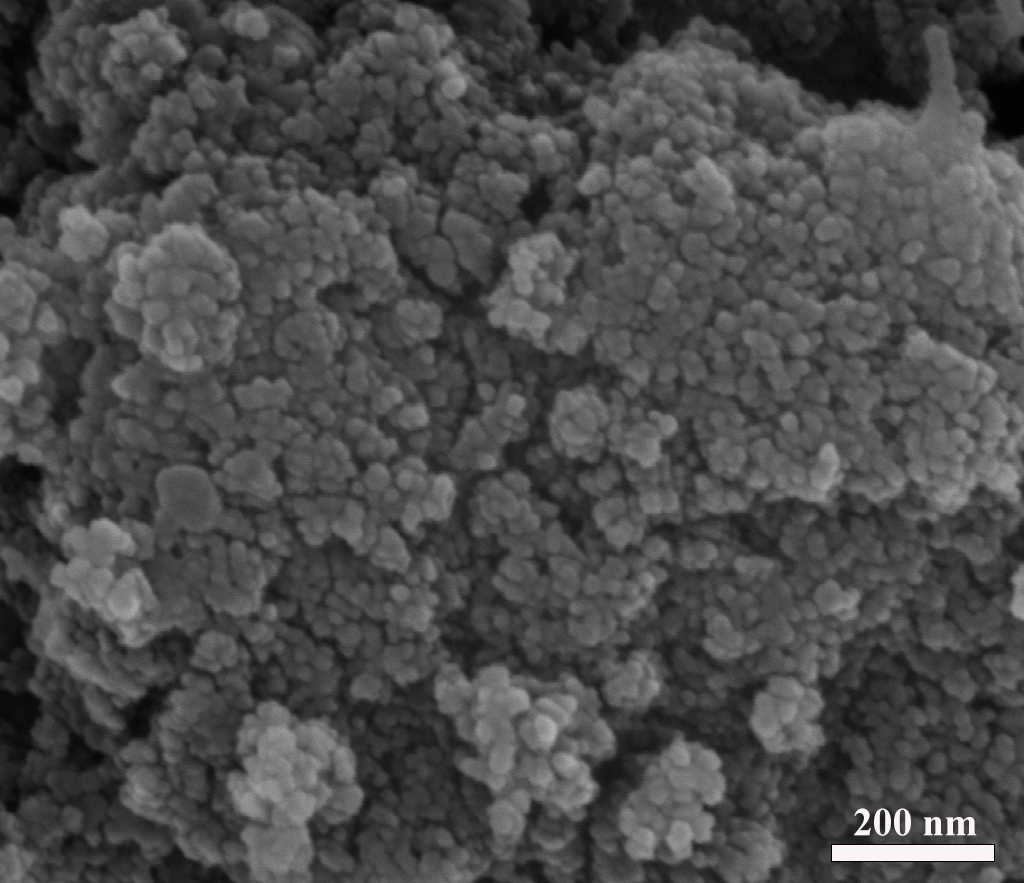

Supplement: S1 Fig — (TIF) [file pone.0304333.s001.tif]

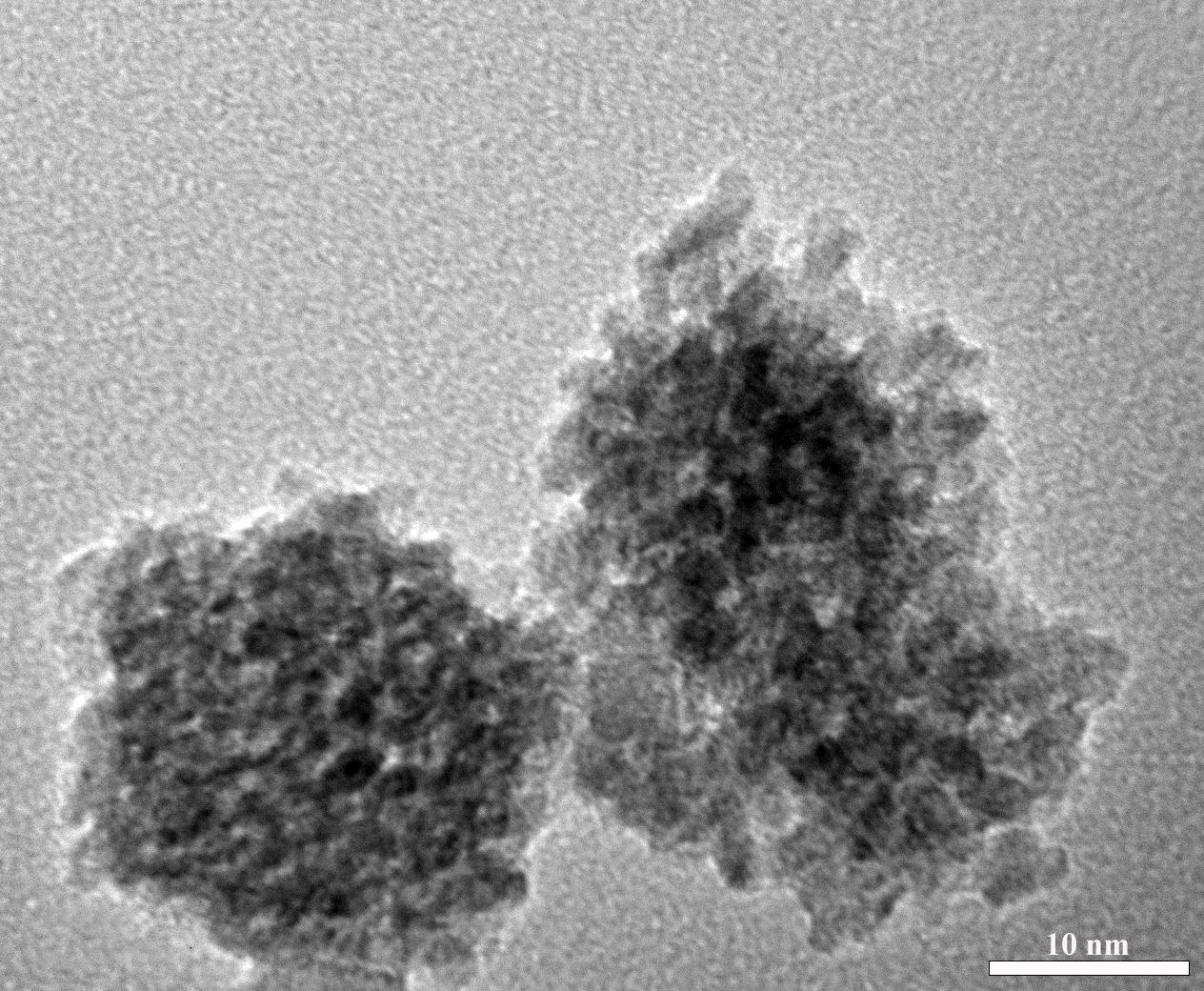

Supplement: S2 Fig — (BMP) [file pone.0304333.s002.bmp]
