# Supplementary material for: Adsorption of magnetic manganese ferrites to simulated monomeric mercury in flue gases
Source: PLoS One. 2024 Jun 14;19(6):e0304333. doi: 10.1371/journal.pone.0304333 (PMC11178181; doi:10.1371/journal.pone.0304333)
Supplement: S1 Table — (DOCX) [file pone.0304333.s005.docx]

**Table S1**. Adsorption data of Hg^0^ by MnFe_2_O_4_ nanoparticles prepared with various alcohol volumes under permeation temperature of 40 °C, space velocity of 4.8×10^4^ h^-1^, and adsorption temperature of 30 °C.

| Group | Alcohol volume (mL) | Absorption capacity (μg/g) | Standard deviation |
| --- | --- | --- | --- |
| 1 | 15 | 1.48 | 0.2 |
| 2 | 20 | 1.72 | 0.27 |
| 3 | 25 | 1.81 | 0.12 |
| 4 | 30 | 5.06 | 0.16 |
| 5 | 35 | 4.89 | 0.23 |
| 6 | 50 | 2.17 | 0.21 |
| 7 | 100 | 1.57 | 0.19 |
