# Supplementary material for: Adsorption of magnetic manganese ferrites to simulated monomeric mercury in flue gases
Source: PLoS One. 2024 Jun 14;19(6):e0304333. doi: 10.1371/journal.pone.0304333 (PMC11178181; doi:10.1371/journal.pone.0304333)
Supplement: S2 Table — (DOCX) [file pone.0304333.s006.docx]

**Table S2**. Adsorption data of Hg^0^ by MnFe_2_O_4_ nanoparticles prepared at different calcination temperatures under permeation temperature of 40 °C, space velocity of 4.8×10^4^ h^-1^, and adsorption temperature of 30 °C.

| Group | Calcination temperature (°C) | Absorption capacity (μg/g) | Standard deviation |
| --- | --- | --- | --- |
| 1 | 400 | 1.48 | 0.1 |
| 2 | 500 | 2.57 | 0.08 |
| 3 | 600 | 1.87 | 0.05 |
| 4 | 700 | 1.58 | 0.11 |
| 5 | 800 | 0.62 | 0.04 |
