# Supplementary material for: Adsorption of magnetic manganese ferrites to simulated monomeric mercury in flue gases
Source: PLoS One. 2024 Jun 14;19(6):e0304333. doi: 10.1371/journal.pone.0304333 (PMC11178181; doi:10.1371/journal.pone.0304333)
Supplement: S3 Table — (DOCX) [file pone.0304333.s007.docx]

**Table S3**. Adsorption capacity of MnFe_2_O_4_ nanoparticles for Hg^0^ at different adsorption temperatures under permeation temperature of 40 °C and space velocity of 4.8×10^4^ h^-1^.

| Group | Adsorbent temperature (°C) | Absorption capacity (μg/g) | Standard deviation |
| --- | --- | --- | --- |
| 1 | 30 | 7.68 | 0.4 |
| 2 | 50 | 16.27 | 0.29 |
| 3 | 80 | 13.22 | 0.24 |
| 4 | 100 | 10.68 | 0.35 |
| 5 | 120 | 8 | 0.22 |
