# Supplementary material for: Adsorption of magnetic manganese ferrites to simulated monomeric mercury in flue gases
Source: PLoS One. 2024 Jun 14;19(6):e0304333. doi: 10.1371/journal.pone.0304333 (PMC11178181; doi:10.1371/journal.pone.0304333)
Supplement: S4 Table — (DOCX) [file pone.0304333.s008.docx]

**Table S4**. Effect of the space velocity on the performance of MnFe_2_O_4_ nanoparticles for Hg^0^ removal under permeation temperature of 40 °C and the adsorption temperature of 50 °C.

| Group | Space velocity (h^-1^) | Absorption capacity (μg/g) | Standard deviation |
| --- | --- | --- | --- |
| 1 | 3.6×10^4^ | 9.45 | 0.13 |
| 2 | 4.8×10^4^ | 16.27 | 0.2 |
| 3 | 6.0×10^4^ | 13.36 | 0.24 |
| 4 | 7.2×10^4^ | 10.13 | 0.17 |
