# Supplementary material for: Adsorption of magnetic manganese ferrites to simulated monomeric mercury in flue gases
Source: PLoS One. 2024 Jun 14;19(6):e0304333. doi: 10.1371/journal.pone.0304333 (PMC11178181; doi:10.1371/journal.pone.0304333)
Supplement: S5 Table — (DOCX) [file pone.0304333.s009.docx]

**Table S5**. Effect of permeation temperature on Hg^0^ removal by MnFe_2_O_4_ nanoparticles under space velocity of 4.8×10^4^ h^-1^ and adsorption temperature of 50 °C.

| Permeation temperature (℃) | 30 | | 40 | | 50 | |
| --- | --- | --- | --- | --- | --- | --- |
| Group | Absorption capacity (μg/g) | Standard deviation | Absorption capacity (μg/g) | Standard deviation | Absorption capacity (μg/g) | Standard deviation |
| 1 | 3.28 | 1.2 | 9.7 | 2.3 | 13.07 | 2.1 |
| 2 | 5.35 | 1.13 | 16.27 | 1.8 | 23.42 | 0.99 |
| 3 | 7.8 | 1.4 | 21.93 | 1.2 | 38.52 | 1.45 |
| 4 | 10.23 | 1.21 | 29.24 | 1.54 | 45.88 | 2.1 |
| 5 | 13.64 | 1.3 | 35.28 | 1.9 | 56.71 | 1.84 |
| 6 | 15.43 | 2 | 40.64 | 2.4 | 66.89 | 1.35 |
| 7 | 17.29 | 1.6 | 47.36 | 1.3 | 75.61 | 2.3 |
| 8 | 21.54 | 0.94 | 55.77 | 2 | 86.28 | 0.89 |
| 9 | 25.72 | 2.1 | 61.73 | 2.5 | 103.3 | 1.23 |
| 10 | 28.72 | 1.29 | 67.73 | 1.59 | 115.04 | 1.6 |
